# Innovative applications of artificial intelligence during the COVID-19 pandemic

**DOI:** 10.1016/j.imj.2024.100095

**Published:** 2024-02-21

**Authors:** Chenrui Lv, Wenqiang Guo, Xinyi Yin, Liu Liu, Xinlei Huang, Shimin Li, Li Zhang

**Affiliations:** a Huazhong Agricultural University, Wuhan 430070, China; b National Institute of Parasitic Diseases, Chinese Center for Disease Control and Prevention; Chinese Center for Tropical Diseases Research, Shanghai 200001, China

**Keywords:** COVID-19, Artificial intelligence, Pandemic prediction, Diagnosis, Drug discovery

## Abstract

The COVID-19 pandemic has created unprecedented challenges worldwide. Artificial intelligence (AI) technologies hold tremendous potential for tackling key aspects of pandemic management and response. In the present review, we discuss the tremendous possibilities of AI technology in addressing the global challenges posed by the COVID-19 pandemic. First, we outline the multiple impacts of the current pandemic on public health, the economy, and society. Next, we focus on the innovative applications of advanced AI technologies in key areas such as COVID-19 prediction, detection, control, and drug discovery for treatment. Specifically, AI-based predictive analytics models can use clinical, epidemiological, and omics data to forecast disease spread and patient outcomes. Additionally, deep neural networks enable rapid diagnosis through medical imaging. Intelligent systems can support risk assessment, decision-making, and social sensing, thereby improving epidemic control and public health policies. Furthermore, high-throughput virtual screening enables AI to accelerate the identification of therapeutic drug candidates and opportunities for drug repurposing. Finally, we discuss future research directions for AI technology in combating COVID-19, emphasizing the importance of interdisciplinary collaboration. Though promising, barriers related to model generalization, data quality, infrastructure readiness, and ethical risks must be addressed to fully translate these innovations into real-world impacts. Multidisciplinary collaboration engaging diverse expertise and stakeholders is imperative for developing robust, responsible, and human-centered AI solutions against COVID-19 and future public health emergencies.

## Highlights

• AI enables COVID-19 forecasting, diagnosis, decision support for response and control.
• Intelligent systems aid risk analysis and policymaking to combat COVID-19.
• AI accelerates drug repurposing and discovery of COVID-19.
• Multidisciplinary collaboration vital responsible AI solutions against COVID-19.

## Graphical abstract

**Figure:**
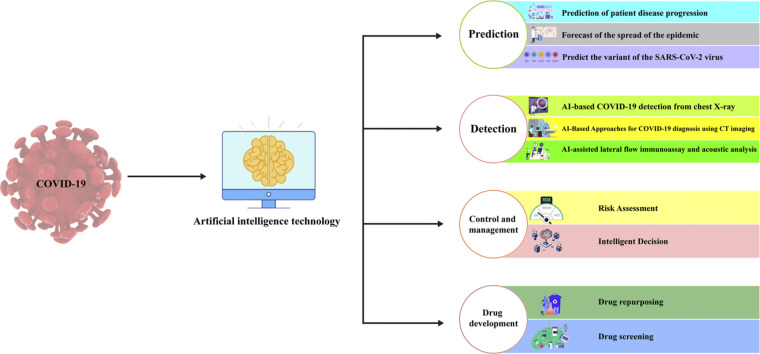

## 1 Introduction

The COVID-19 pandemic caused by the SARS-CoV-2 coronavirus has created unprecedented global challenges spanning public health, economics, and society [1,2]. Since its emergence in late 2019, the highly infectious virus has led to over 620 million confirmed cases and more than 6.5 million deaths as of October 2022 [3]. Beyond the staggering direct impacts, the pandemic has severely disrupted international travel, supply chains, education, and health care systems. The resultant economic losses and societal burdens continue to be felt across the world [4], [5], [6].

To curb the spread of COVID-19 and mitigate its multi-faceted impacts, governments have implemented various containment and mitigation measures, including lockdowns, mask mandates, social distancing rules, mass testing, and vaccination drives [7,8]. However, the rapidly evolving nature of the pandemic has strained the capacities of public health systems and challenged traditional response strategies [9], [10], [11]. Innovative technological solutions have become imperative to tackle the unique complexities of this public health crisis in a timely and effective manner [12].

Artificial intelligence (AI) has emerged as a promising tool, offering data-driven solutions to address major challenges in managing the pandemic [13,14]. Advanced machine learning and deep learning techniques can unlock insights using large-scale datasets related to coronavirus transmission, disease progression, patient outcomes, population mobility, and health care operations [15], [16], [17]. AI-based predictive analytics, intelligent diagnosis, risk assessment systems, decision support platforms, and computational drug discovery hold tremendous potential for improving the prediction, detection, control, and treatment of COVID-19 [14,[18], [19], [20], [21]]. However, to translate proofs of concept into real clinical and public health impact, technological innovations must be paired with a nuanced understanding of relevant epidemiological, clinical, ethical, and social contexts.

**Fig. 1:**
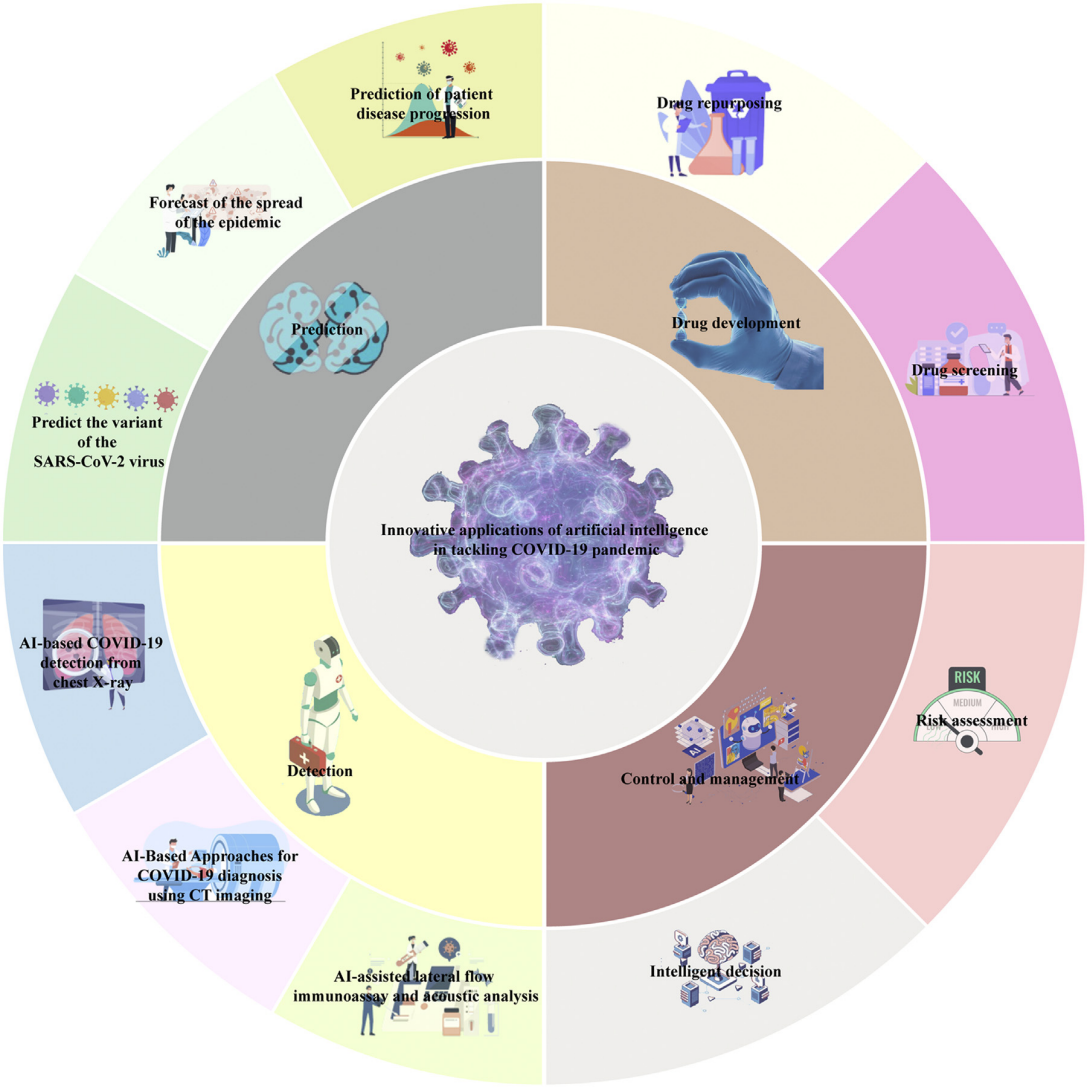
Applications of artificial intelligence (AI) in the COVID-19 pandemic.

In this review, we summarize state-of-the-art applications of AI methodologies in combating COVID-19, highlight key enabling factors as well as limitations, and discuss directions for future research. In the following sections, we discuss AI applications in COVID-19 control and management, drug development, and other related domains (Fig. 1). Intelligent systems incorporating risk assessment, decision support, and social sensing have been developed to aid public health responses. Machine learning and deep learning models have accelerated the discovery of anti-COVID-19 therapeutics via high-throughput screening and drug repurposing. In this review, we aim to provide an overview of state-of-the-art AI techniques that are transforming the battle against the COVID-19 pandemic. By leveraging the power of AI, we can continue to effectively combat the challenges posed by the ongoing pandemic and pave the way for a healthier future.

## 2 General introduction to AI

AI refers to the capability of computer systems to execute functions that typically necessitate human intelligence, such as visual perception, speech recognition, decision-making, and language translation [22,23]. The fundamental objective of AI is to enable machines to learn from data and experience, thereby enabling them to effectively and adaptively perform tasks resembling those carried out by humans. Machine learning, a subset of AI, facilitates algorithms in learning from data without explicit programming. Instead of relying solely on hard-coded rules, machine learning algorithms construct predictive models by identifying patterns within extensive datasets [24]. As more data are incorporated, these models continuously update to enhance their performance in specified tasks such as classification, regression, and clustering. Commonly used machine learning algorithms include linear regression, logistic regression, naive Bayes classifiers, the k-nearest neighbors algorithm, support vector machines, decision trees (DTs), and neural networks, each with its own strengths, weaknesses, and application scenarios [25,26]. Selection of the most suitable machine learning technique is crucial to harnessing the potential of AI in various fields. As research advances, new and hybrid algorithms are being actively developed and applied to address real-world problems [27].

Deep learning is an AI technique that emulates the neural networks of the human brain [28]. Deep learning uses multi-layered neural networks to extract high-level abstract features from data, enabling pattern recognition and predictive analysis. In comparison with traditional machine learning methods, deep learning excels in domains such as image, speech, and text processing. Deep learning has greatly contributed to COVID-19 pandemic forecasting [29]. For instance, early warning systems developed using deep learning algorithms can analyze clinical data and computed tomography (CT) scans to predict the deterioration of patients with COVID-19 in advance, thereby aiding physicians in making well-informed treatment decisions [30,31].

Neural networks are computational models that simulate the connections and functioning principles of neurons in the human brain [32]. They consist of interconnected processing units and establish nonlinear mapping relationships between inputs and outputs through learning from training datasets. Unlike traditional algorithms, neural networks can learn complex data patterns and exhibit strong adaptability, performing exceptionally well in handling high-dimensional data such as images, speech, and text [33]. In prediction during the COVID-19 pandemic, neural network models have played a pivotal role. The potent nonlinear expressive capability of neural network models lends a significant advantage in addressing complex problems like pandemic forecasting, making them a key technological approach in this field [34,35].

The decision tree model is a widely used machine learning algorithm with diverse applications in prediction and classification problems [36]. These models utilize a tree representation to partition a dataset and derive decisions based on features. Although these models are interpretable, they can easily overfit. Random forests improve performance by averaging predictions from multiple trees. By analyzing and determining features, the DT represents the decision-making process in a tree-like structure, facilitating accurate predictions and decisions [37,38]. In the context of the COVID-19 pandemic, the application of DTs holds great importance in predicting patient hospitalization time, mortality risk, and other critical indicators.

The long short-term memory (LSTM) network is a recurrent neural network (RNN) commonly applied for time-series prediction [39]. LSTM has the capability to learn long-term dependencies in time-series data and use them in forecasting [40]. Within the context of the COVID-19 pandemic, LSTM has been applied to model the time series of confirmed cases and predict future trends [41]. In comparison with other forecasting models, LSTM demonstrates superior accuracy in capturing complex patterns within time-series data, thereby making it highly valuable for epidemic prediction [42,43].

## 3 AI for COVID-19 prediction

### 3.1 Prediction of patient disease progression

**Fig. 2:**
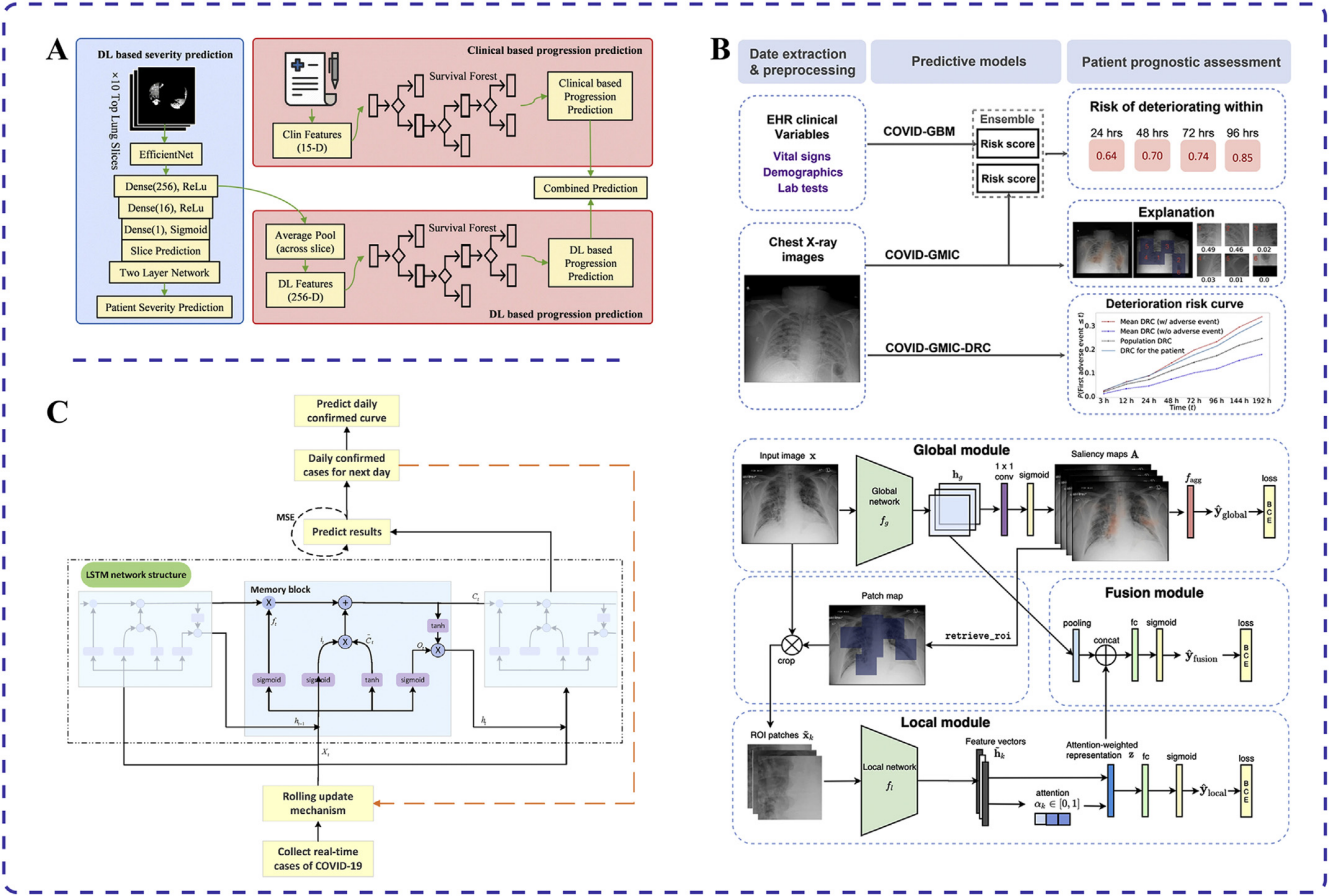
Applications of different artificial intelligence (AI) techniques for COVID-19 prediction. (A) Deep learning AI system using chest computed tomography images and clinical data to predict disease progression in patients with COVID-19 [44]. (B) Combination of deep neural networks and gradient boosting models used to assess the risk of disease progression and deterioration in patients with COVID-19 through analysis of chest X-ray images [46]. (C) Long short-term memory algorithm augmented with an embedded rolling update mechanism for long-term prediction of COVID-19 cases [47].

Recent advancements in AI have paved the way for various applications in predicting disease progression and outcomes in patients with COVID-19. Specifically, deep learning AI systems based on chest CT images and clinical data have been adopted to predict COVID-19 disease progression [44,45]. Wang et al. used CT image segmentation techniques to screen pneumonia lung tissues and identify abnormalities in the lung parenchyma from COVID-19-positive patients [44] (Fig. 2A). Those authors built severity and disease course prediction models using deep learning to anticipate progression to adverse outcomes such as admission to the intensive care unit, use of mechanical ventilation, or death. The concordance indices for these models were 0.719 and 0.774, respectively, indicating their importance for timely intervention in patient care to reduce mortality [44].

Researchers have also developed early warning systems using deep learning algorithms with collected clinical data and patient CT scans to predict deterioration processes in patients with COVID-19 [48,49]. By successfully integrating clinical data and CT images, Fang et al. validated an area under the receiver operating characteristic curve (AUC) of 0.920 for this method in a single-center study, compared with 0.851 and 0.787 for clinical data or CT images alone, respectively [49]. The AUC is a vital metric for evaluating the performance of binary classification models. It represents the area under the receiver operating characteristic curve, which plots the true positive rate against the false positive rate at different classification thresholds. AUC values range from 0 to 1, with higher values indicating better model classification performance. Generally, an AUC above 0.5 suggests some classification ability whereas values above 0.8 indicate good performance, and those above 0.9 signify exceptionally high performance. In summary, the AUC reflects a model's ability to correctly classify positive and negative samples across different thresholds. The system can also identify key indicators of worsening conditions, such as biomarkers like troponin, brain natriuretic peptide, and white blood cell count [49]. This helps physicians determine the treatment priorities for patients to optimize therapeutic strategies.

An AI system has also been developed to predict the deterioration trend of patients with COVID-19 pneumonia in the emergency department [46]. Shamout et al. used chest X-ray images and routine clinical variables to predict the trend of deterioration using a data-driven approach that combines deep neural networks and gradient boosting models (Fig. 2B). When predicting the deterioration trend within 96 hours for 3661 patients, the model achieved an AUC of 0.786. This method provides clinicians with a quantitative estimate of the risk of deterioration, helping to prioritize and treat high-risk patients [46].

With the capabilities of AI models, it is possible to accurately predict the hospitalization time and mortality risk of patients with COVID-19 [50,51]. Mahboub et al. collected clinical data of COVID-19 cases from the Dubai Health Authority in 2019 and used multivariate analysis to identify key variables [52]. Subsequently, they built a DT using these key variables to predict patient hospitalization time and mortality risk. The model achieved a determination coefficient of 49.8% for predicting hospitalization time and an accuracy rate of 96% for predicting mortality risk. These results are of great importance for clinicians to strengthen treatment plans and management strategies, enabling the rational allocation of resources [52].

In another study, a probability model was developed to predict whether patients with COVID-19 would develop acute respiratory distress syndrome (ARDS) by analyzing the clinical characteristics of patients with ARDS [53]. The researchers compared clinical data of patients with and without ARDS and used machine learning and deep learning algorithms to analyze clinical features related to ARDS, such as cough, dyspnea, lung consolidation, and secondary bacterial infections. Additionally, they constructed a DT using patients' biochemical indicators to determine whether patients without ARDS would develop ARDS [53]. The model demonstrated good sensitivity and specificity, with an accuracy rate of 91%, surpassing the average accuracy rate of other models in the same category (70%). This research contributes to alleviating the global shortage of ventilators caused by ARDS.

Another algorithm was developed to rapidly identify patients with COVID-19 at high risk of rapid death [54]. Yang et al. analyzed the clinical features and laboratory indicators of patients with severe and non-severe COVID-19 as well as patients who survived and those who died [54]. They found significant differences in the neutrophil-to-lymphocyte ratio, C-reactive protein, and lactate dehydrogenase among the different groups. Based on these indicators, they developed a DT to identify high-risk patients with COVID-19. The model could accurately predict the mortality rate of critically ill patients with a precision of 98%. This aids in prioritizing the treatment of high-risk patients [54]. Similarly, an AI statistical approach developed by Sinha and Rathi analyzes exploratory factors such as age and sex to predict the likelihood of survival in patients with COVID-19 [55]. The researchers constructed a model using hyperparameter-tuned machine learning and deep learning based on autoencoders to assess the impact of different factors on the survival rate of patients in isolation and conduct prediction. With an average accuracy of 91% in predicting deaths according to logistic regression analysis, such predictive models can analyze critical time points in the spread of an epidemic to assist in national policy planning [55].

### 3.2 Forecasting epidemic size or COVID-19 trends

Continued advancement in customized predictive solutions that incorporate expanded data sources may further aid pandemic response efforts. One effective forecasting model for daily COVID-19 cases worldwide combines the auto regressive integrated moving average (ARIMA) model and neural network approaches [56]. Initially, the authors used the ARIMA model to predict daily COVID-19 cases globally in 2021 [56]. However, owing to its inability to capture the nonlinear structure of the data, the model had low computational intensity. To overcome this limitation, the authors adopted a multilayer perceptron (MLP) neural network to analyze the residuals of the ARIMA model, enabling nonlinear analysis. This MLP-ARIMA hybrid model can make accurate predictions in advance by considering dynamically updated data, thereby reducing the number of new cases [56]. However, it is worth noting that this model does not account for the impact of data mutations, although it can still produce satisfactory results over an extended period under certain control conditions.

Another study used an improved chaotic marine predators algorithm-adaptive neuro-fuzzy inference system (CMPA-ANFIS) model to predict confirmed COVID-19 cases in Brazil and Russia [57]. The authors used an ANFIS to establish a short-term prediction model and enhance it with the marine predation algorithm (MPA). Additionally, chaos mapping was applied to the MPA to improve its balance detection and exploitation stage performance. Taking the prediction in Russia as an example, the CMPA model achieved lower root mean square error/mean absolute error/mean absolute percentage error/root mean square relative error values (493/379/0.03223/0.0004) than ANFIS, particle swarm optimization, and MPA models [57]. The CMPA-ANFIS model can predict the total number of COVID-19 cases in a specific region and assist decision-makers in formulating epidemic prevention plans.

The establishment of accurate prediction models holds crucial importance in forecasting COVID-19 cases. Sinha et al. applied artificial neural networks (ANNs) and LSTM, a type of RNN, for prediction and conducted model testing in five different countries [58]. Taking France as an example, the mean squared errors for the LSTM and ANN models were 0.0044 and 0.0140, respectively. The results demonstrated that the LSTM model yielded fewer prediction errors than the ANN model across all countries, indicating a higher level of prediction accuracy. By optimizing the model, better epidemic predictions can be achieved, guiding health care professionals in the rational allocation of medical resources for effective prevention and control [58].

Researchers have developed a hybrid prediction model for COVID-19 transmission at various time points [59]. Building upon traditional models, they incorporated an improved–susceptible–infected model, a natural language processing module, and an LSTM network to enhance prediction accuracy. The study revealed that the period between days 3 and 8 after infection is when patients are most likely to transmit the virus, aligning with real-world observations. This model significantly reduces errors, with average absolute percentage errors of only 0.52%, 0.38%, and 0.03% for prediction in Wuhan, Beijing, and Shanghai, respectively. By considering the impact of prevention and control measures, this model highlights the crucial role of information disclosure in epidemic prevention and control [59].

An improved LSTM deep learning model has been developed to predict the trend of COVID-19 outbreaks [47]. Wang et al. enhanced traditional statistical models by incorporating an embedded rolling update mechanism into the LSTM algorithm, enabling long-term predictions (Fig. 2C). They also included diffusion index analysis to evaluate the effectiveness of prevention and control measures. The model achieved an average error of only 1.43% when predicting confirmed cases in Russia from July 8 to July 11 in 2020, closely matching the actual development of the epidemic in that country [47]. This model provides valuable support for government decision-making in formulating prevention and control plans.

### 3.3 Predicting variants of the SARS-CoV-2 virus

The emergence of new viral variants of SARS-CoV-2, characterized by increased immune evasion, transmissibility, and pathogenicity, has compounded the challenges in controlling the COVID-19 pandemic [60,61]. Therefore, it is crucial to effectively predict these variants. Ullah and his team employed a convolutional neural network (CNN) for classifying COVID-19 variants [62]. The researchers used one-dimensional CNNs, batch normalization, and self-attention layers to identify sequence relationships and mutations in adenine, cytosine, uracil, and thymine within SARS-CoV-2 nucleic acids. By predicting variant strains based on given nucleotide sequences of SARS-CoV-2, they applied a variational autoencoder–decoder network and used the Basic Local Alignment Search Tool (BLAST) to determine whether the variant originated from existing strains or represented a novel mutation [62]. This approach of predicting nucleotide sequences of variant strains can assist vaccine manufacturers in enhancing vaccine quality.

The rapid global spread of the Omicron variant of SARS-CoV-2 and its increased transmissibility have resulted in a higher mortality rate, severely affecting the effectiveness of global vaccination programs [63]. The spike protein, a glycoprotein on the virus envelope, is involved in attachment and invasion of the virus in host cells [64]. Nagpal et al. proposed a system called Strainflow, which carries out genomic surveillance of COVID-19 spike protein sequences [65]. The system splits the sequence codons and uses supervised and causal prediction models based on unsupervised latent space features to estimate novel variants associated with spike protein mutations. By analyzing 900,000 spike protein gene sequences and applying the random forest regression algorithm, the model could successfully capture the increase in case numbers 2 months before the surges caused by the Delta and Omicron variants [65]. This system can help mitigate the reduced vaccine efficacy caused by SARS-CoV-2 variants by predicting variant strains.

The emergence of SARS-CoV-2 mutations can impact the virus's infectivity and the effectiveness of vaccines. Predicting variant strains that can evade immune detection is crucial for vaccine development. Thadani et al. developed EVEscape, a deep learning generative model trained on historical virus sequences, combined with structural and biophysical constraints [66]. This model can predict mutation pathways of the virus without relying on the latest virus sequences and antibody information. The model is suitable for the early stages of COVID-19 development and enables continuous assessment of newly emerging variants [66]. This method achieves prediction accuracy similar to that of high-throughput experimental scanning, anticipates mutations months ahead of antibody and serum tests, and can also be applied to predicting variations in other influenza and pandemic viruses.

## 4 AI for detection of COVID-19

### 4.1 AI-based COVID-19 detection using chest X-ray

CNNs have emerged as crucial tools in detecting COVID-19 using chest X-ray, offering a powerful means for early detection and diagnosis of the disease [67,68]. Consequently, CNNs have an important role in hospital infection control and patient care. One example of AI applications in COVID-19 detection is the use of deep learning models to analyze chest X-ray scans [69]. Vaid et al. developed a deep learning model based on the CNN algorithm, which can detect abnormalities and classify diseases directly from chest X-ray scans. To enhance the detection capability of the CNN, transfer learning was adopted by examining patients’ anterior and posterior chest images. The model achieved 96.3% accuracy and can be used for direct chest X-ray testing, eliminating the need for radiologists to perform secondary examinations [69].

The DeepCOVID-XR algorithm is a method specifically designed for identifying COVID-19 from chest X-ray [70]. It was developed by Wehbe et al. and utilizes the results of reverse transcription polymerase chain reaction (RT-PCR) as an indicator [70]. The algorithm uses an ensemble of CNNs to analyze chest X-ray images. The dataset images are preprocessed and four images are generated from each, which are then fed into six CNN frameworks: densenet-121, ResNet-50, Inception-v3, Inception-ResNetV2, Xception, and EfficientNet-B2. By fine-tuning the input set through transfer learning, the predictions from these six frameworks are combined using a weighted average to evaluate whether the image indicates COVID-19 positivity or negativity [70]. This study surpasses similar research in terms of both dataset quantity and quality, with positive implications for hospital infection control.

Computer-aided diagnosis systems can analyze chest X-ray to identify COVID-19 infections. The system initially uses a Unet to extract the region of interest on lung images, obtaining binary lung maps [71]. Image preprocessing methods are then applied to enhance rotation. The segmented images are combined with a CNN model and a transformer vision model. The CNN model uses pixel arrays, and the transformer vision model divides the image into visual tokens. The CNN-based EfficientNet-B7 (with an accuracy of 99.82%) and the vision-based SegFormerB5 (with an accuracy of 99.81%) perform comparably. Furthermore, this system enables the visualization of areas of infection on chest X-ray images, facilitating better care provision and expediting the patient recovery process [71].

Researchers have proposed a method for detecting COVID-19 using chest X-rays based on an intelligent deep convolutional network [72]. Alshahrni et al. combine deep CNNs, integrated bootstrap aggregating neural networks, and multiple neural network clustering methods to enhance diagnostic sensitivity and reduce error rates. They used the TSEBANN model to investigate the effect of qualification procedures. The algorithm classifies examples through preprocessing, feature extraction, and CNN strategies, and it has been tested on a COVID-19 X-ray dataset, which confirmed its effectiveness through cross-validation with a classification accuracy of 98.062%. Compared with chest CT, this method is more cost-effective [72].

A deep CNN based on homomorphic transformation and a visual geometry group has been developed for detecting COVID-19 from chest X-ray images [73]. This method uses contrast-limited adaptive histogram equalization and homomorphic transformation filtering for pixel-level processing of X-ray images. It solely relies on single-channel image data as input, which reduces the complexity of three-channel operations required with RGB image datasets. The processed information is then fed into a deep CNN based on a visual geometry group for analysis, ultimately classifying the images into three categories: normal, COVID-19, and pneumonia. The model can achieve a multi-class classification accuracy of up to 98.06% [73]. This method is simple and fast, serving as a key tool in the detection of COVID-19 from chest X-ray. It provides robust support for the early detection and diagnosis of COVID-19, with a positive role in hospital infection control and patient care.

**Fig. 3:**
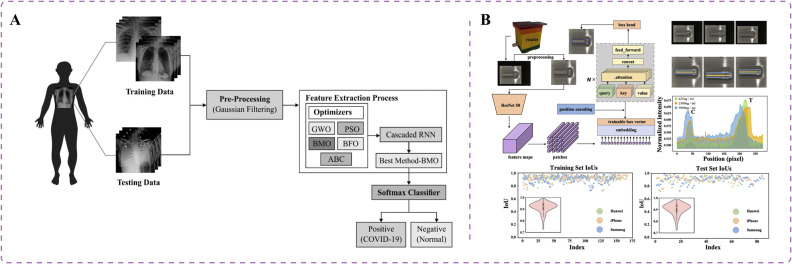
Innovative artificial intelligence approaches for COVID-19 detection. (A) Intelligent COVID-19 diagnostic model, based on the barnacle mating optimization algorithm and cascaded recurrent neural network model, aids in disease diagnosis using low-contrast X-ray images [75]. (B) Deep learning approach using visual transformers developed to create a lateral flow immunoassay platform for smartphones, enabling colorimetric detection of neutralizing antibodies against SARS-CoV-2 in serum [79].

Technological advancements have a crucial role in the early screening and diagnosis of COVID-19, enabling health care professionals to effectively identify and categorize the disease. Recent studies have investigated the potential of RNNs in detecting COVID-19 across different modalities [74], [75], [76]. RNNs, a specific type of ANN, have been designed to handle sequential data. Unlike conventional feedforward neural networks, RNNs incorporate feedback connections that facilitate information propagation within the network [77]. In COVID-19 diagnosis, chest X-ray and CT scans are crucial for assessing patients' health conditions. However, chest CT screening involves greater radiation exposure and is more expensive whereas traditional X-ray machines are convenient and portable in clinic settings [78]. Shankar et al. introduced an intelligent COVID-19 diagnostic model based on the barnacle mating optimization (BMO) algorithm and cascaded recurrent neural network (CRNN) model [75] (Fig. 3A). The CRNN model has been applied in feature extraction from chest X-ray images, followed by the BMO algorithm for hyperparameter optimization of the CRNN to enhance classification performance. The BMO algorithm can determine the optimal values of CRNN hyperparameters, including learning rate, batch size, activation function, and epoch count. The resulting model achieved an average sensitivity of 97.01%, specificity of 98.15%, accuracy of 97.31%, and F-measure of 97.73% [75]. This research contributes to the diagnosis of diseases by radiologists using low-contrast X-ray images.

### 4.2 AI-based approaches to COVID-19 diagnosis using CT imaging

AI has been used to differentiate between COVID-19 and community-acquired pneumonia (CAP) using chest CT scans [80]. Researchers developed a deep learning model called COVNet, which takes CT slices as input and extracts both two-dimensional and three-dimensional global features from chest CT scans. These features are combined using max pooling and passed through fully connected layers and a softmax activation function to generate probabilities for COVID-19 and CAP. The model achieved an AUC of 0.96 for COVID-19 prediction and 0.95 for CAP prediction, effectively distinguishing between the two conditions [80].

To reduce radiation exposure during examinations, ultra-low-dose CT (ULDCT) imaging has been similarly used for COVID-19 diagnosis. However, the low dose of CT images leads to decreased accuracy and requires more time for COVID-19 classification [81,82]. Kannan et al. applied an attention segmental recurrent neural network (ASRNN) to detect patients with COVID-19 using ULDCT images [83]. First, they extracted radiomic features such as morphology, grayscale statistics, and Haralick textures from CT images using generalized additive models with structured interactions. The ASRNN classifier, optimized with the Archimedes optimization algorithm, could classify ULDCT images as patients with COVID-19 or normal individuals. The F-score of the proposed method was 86.43% higher than the Multiple Kernels-ELM-based Deep Neural Network model reported by Turkoglu [83,84].

A multi-task deep learning approach enables the rapid identification of patients with COVID-19 [85]. Alom et al. used a residual recursive CNN based on transfer learning for pneumonia detection and their NABLA-N network model for infection region segmentation [85]. This method not only permits qualitative diagnosis of patients who have COVID-19 with 87.26% accuracy but it also facilitates quantitative analysis of the extent of infection areas based on CT images, achieving accuracy of 98.78%.

### 4.3 AI-assisted lateral flow immunoassay and acoustic analysis

AI-assisted lateral flow immunoassay with colorimetry can be used to detect neutralizing antibodies in patients with COVID-19 infection. Neutralizing antibodies can bind to the virus and eliminate its infectivity [86]. Researchers applied the deep learning method of vision transformers to assist lateral flow immunoassay with polydopamine-based colorimetric detection [79]. They developed a lateral flow immunoassay platform integrated with a smartphone-based reader for quantitative detection of neutralizing antibodies in serum (Fig. 3B). The algorithm has a detection range of 625–10,000 ng/mL. The polydopamine-based colorimetric detection method exhibits strong absorption in the visible light region, enhancing the sensitivity of detection. This approach can be adopted to effectively evaluate the immune response of vaccine recipients, contributing to the establishment of herd immunity [79].

Hassan et al. proposed a method for early screening and diagnosis of COVID-19 based on language signals [76]. They used a RNN with an LSTM framework to analyze the acoustic features of patients' cough and respiratory sounds. Performance of the model in detecting COVID-19 based on cough and respiratory sounds was evaluated in terms of accuracy, recall rate, and AUC. The results showed an accuracy of 98.2% for respiratory sounds and 97% for cough sounds. The research team aims to improve the accuracy of the speech test by expanding the sample set [76].

## 5 Control and management

### 5.1 Risk assessment

In recent years, various systems and technologies have been developed to address COVID-19 risk assessment. These advancements use AI algorithms and techniques to dynamically assess the risk of COVID-19 transmission and infection, assist health care professionals in data collection and risk assessment, determine social distancing compliance, and leverage cloud and fog computing for efficient monitoring and control [87,88].

One notable system is the α-Satellite system, which is used to dynamically assess the risk of COVID-19 in the United States [89]. This system automatically generates a layered risk index based on user-input points of interest (POIs). Initially, the system establishes a database and uses an attribute heterogeneous information network (AHIN) to model the data [89]. Subsequently, performance of the AHIN is enhanced through the generation of conditional improvements using generative adversarial networks (GANs), resulting in a diverse COVID-19 risk assessment system. Upon validation with 5060 real POIs, the system achieved an AUC of 0.9202. This system can aid in selecting appropriate protective measures to mitigate viral spread and infection.

The DDC19 system has been used to assist health care professionals in data collection and dynamic risk assessment during the COVID-19 pandemic [90]. This system combines the medical process with designed flowcharts and questionnaires. By evaluating the questionnaires, the system establishes a dynamic risk stratification model and enhances the modelʼs accuracy through regression analysis [90]. The model demonstrates strong predictive capabilities for risk levels in various scenarios.

**Fig. 4:**
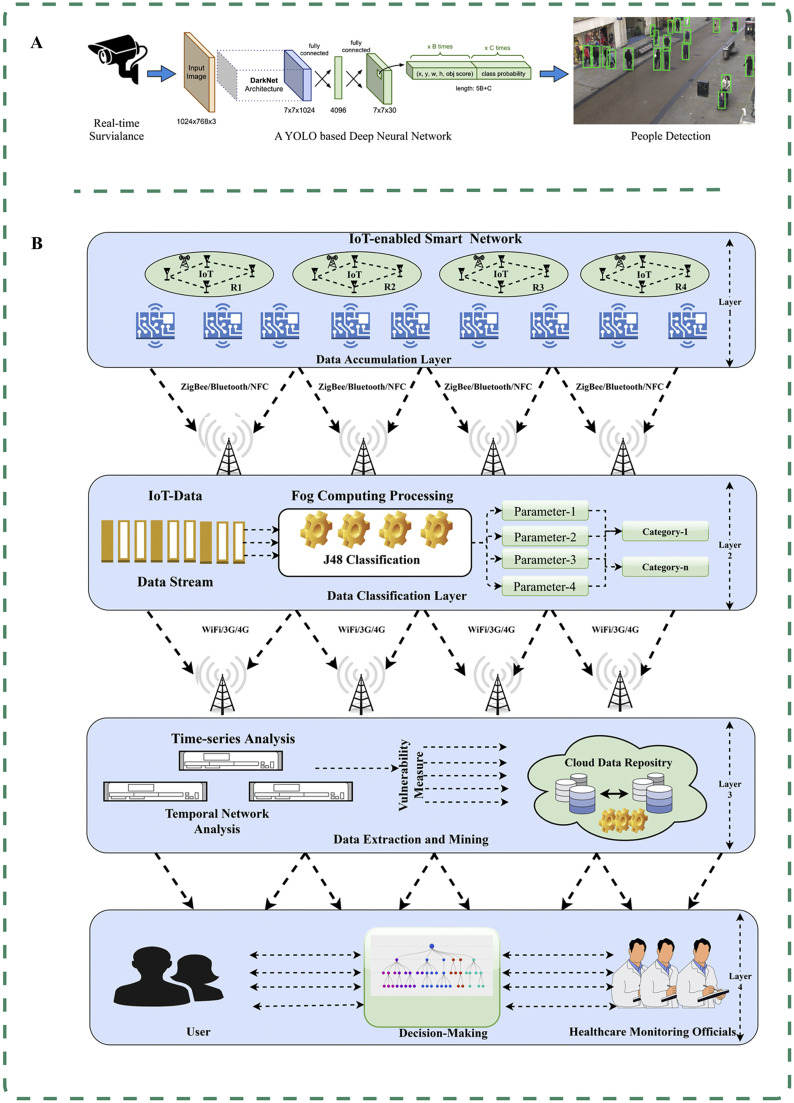
Artificial intelligence-empowered control and management of COVID-19. (A) DeepSocial enables automatic monitoring of indoor and outdoor crowds and social distancing to identify areas with the highest likelihood of virus transmission and infection [91]. (B) Combining radio frequency identification devices with fog computing in the Internet of Things (IoT), and capturing social interactions and physical symptoms to classify users [87].

Rezaei and Azarmi developed a hybrid computer vision and Yolov4-based deep neural network model for detecting persons and social distancing detection [91]. The model integrates adaptive inverse perspective mapping technology and the SORT tracking algorithm to automatically detect crowds in indoor and outdoor environments (Fig. 4A). Through extensive testing, the model achieves an average precision of 99.8%, enabling the identification of areas with the highest likelihood of virus transmission and infection. This capability has proven highly beneficial for government agencies in designing layouts and implementing preventive measures in public spaces [91].

Cloud and fog computing offer a cost-effective and time-saving approach to monitoring and controlling the rapid spread of infections. Singh and Kaur combine cloud and fog computing with a classifier that integrates random forest, naive Bayes, and GANs for COVID-19 classification [92]. They proposes a service quality framework based on fog-assisted IoT, which collects sensitive information from connected devices and responds by issuing alerts to relevant departments for action [92].

### 5.2 Intelligent decision-making

The COVID-19 pandemic has presented unparalleled challenges to global health care systems, necessitating innovative and effective approaches to combat spread of the virus [93]. AI has emerged as a potent tool in the fight against COVID-19, offering new possibilities for policy decision-making [9,94]. For instance, a novel AI-based technology has been developed to identify and remotely monitor patients, combining wireless radio frequency identification (RFID) devices with fog computing in the IoT [87]. This innovative approach enables efficient patient identification and management, using RFID to capture social interactions and using IoT technology to gather information on physical symptoms (Fig. 4B). Bhatia et al. applied a DT to assess infection severity and classify users based on their corresponding symptoms, achieving an impressive accuracy rate of 96.68% and enabling effective and timely monitoring [87].

In the context of disease outbreaks, an AI algorithm can be applied to formulate policies for reopening schools [95]. This algorithm comprises a multi-input multi-output model with uncertainty and adaptive background parameter modeling. It establishes an adaptive background single-input multiple-output parameter model and follows an adaptive strategy for external manipulation to generate appropriate policies. The model's long-term predictor evaluates the potential impacts under the current policy, and the policy maker proposes alternative measures to minimize these impacts. By leveraging this algorithm, policymakers can formulate effective policies aimed at minimizing COVID-19 mortality and maximizing school enrollment [95].

The Epidemic and Media Impact Tool (EMIT), an advanced technology, leverages COVID-19-related information from social media for outbreak detection and control. Lazebnik et al. proposed a dual-model approach consisting of a next-wave predictor and a social epidemiological simulator, which is used for training and inference [96]. In the training phase, machine learning techniques are used to fit the parameters of the next-wave predictor and the simulator. In the inference phase, the latest data are used for prediction and computation of baseline predictions of widespread transmission. The EMIT provides predictive estimation regarding the impact of historical data, social media trends, and disease transmission data on public health measures, achieving an impressive AUC of 0.909 [96].

A top-down multiscale engineering approach can effectively determine optimal control measures implemented by regulatory agencies during the COVID-19 pandemic [97]. This approach involves global-scale modeling of COVID-19 growth rates and mortality rates, supplemented by deep neural networks that consider multiple time steps in the prediction modeling step. The SHapley Additive exPlanations method is used to identify the most influential control factors. These identified factors greatly contribute to reducing epidemic growth rates and mortality rates. Based on this study, it is concluded that covid-contact-tracing, public-gathering-rules, and covid-stringence-index are the three most effective control measures for minimizing growth rates. The control factors associated with mortality rates depend on the specific modeling scenario [97].

Levashkin et al. developed an intelligent decision-making system to study the impact of decisions in the context of COVID-19 [98]. Initially, they constructed the Awareness–Compartmental Model-Susceptible–Exposed–Infectious–Removed (ACM-SEIR) model based on the SEIR model, incorporating parameters such as risk perception and cumulative case count. Machine learning techniques were then used to investigate the model, and the ACM-SEIR parameter module was adjusted using ontology-based association methods to transform it into an intelligent decision-making system. This advanced model allows for the evaluation of social, economic, and other factors in decision-making processes [98].

## 6 Drug development

### 6.1 Drug repurposing

Given the complexity of drug design and clinical trials, repurposing existing drugs is crucial in the search for COVID-19 treatments. Mohapatra et al. applied machine learning models to a dataset selected from PubChem [99,100]. To enable the system to learn from datasets containing details and practical outcomes, they adopted mathematical classifiers for supervised learning. The naive Bayes classifier was found to be the optimal choice, avoiding overfitting issues encountered with random forest or sequential minimal optimization algorithms [99,100]. The model achieved an accuracy of approximately 73% in drug prediction. Ultimately, they determined that the antiretroviral drug amprenavir was the most effective against COVID-19 infection.

**Fig. 5:**
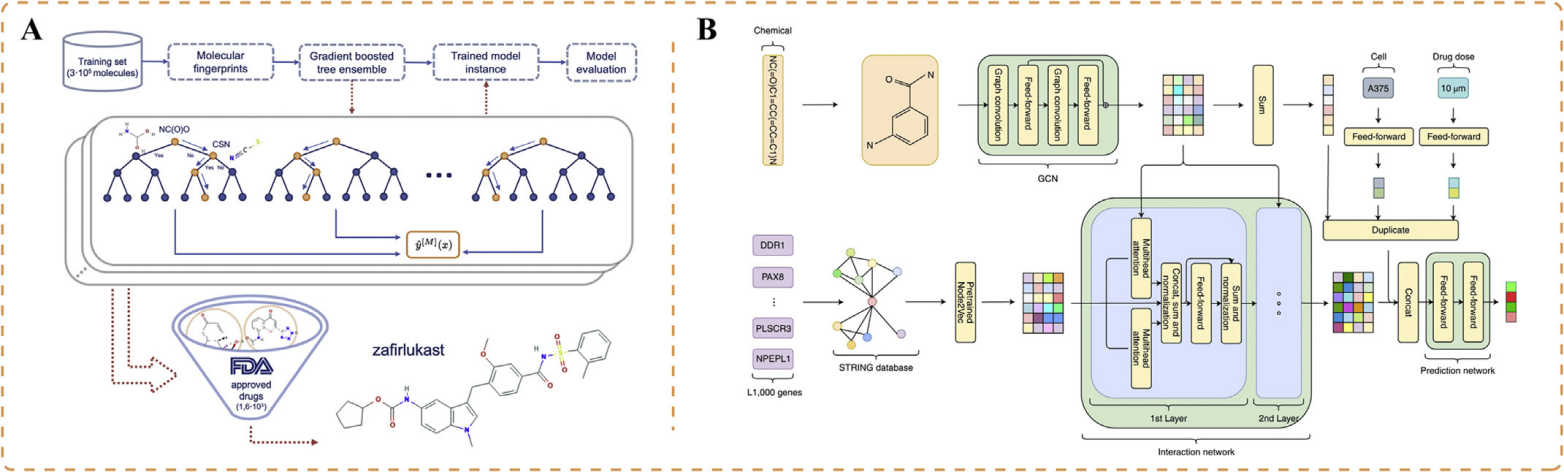
Applications of artificial intelligence for COVID-19 drug repurposing. (A) Machine learning model that uses extracellular data encoded with chemical fingerprints to partition molecular fingerprints using a collection of individual tree models to uncover drug molecule characteristics and predict potential candidate drugs [101]. (B) Mechanism-driven neural network approach (DeepCE) that predicts the impact of new chemical entities on differential gene expression profiles by simulating the relationships between chemical substructures and genes, as well as gene–gene interactions [102].

Researchers have conducted drug-based prediction of antiviral activity against COVID-19 to identify potential candidates for drug repurposing. Delijewski and Haneczok developed a supervised machine learning model that uses in vitro data encoded with chemical fingerprints, representing specific molecular substructures [101]. They adopted a sequence ensemble of individual tree models to partition the molecular fingerprints and uncover the molecular properties of various candidate drugs (Fig. 5A). By updating the tree models, they improved the model, resulting in an AUC of 0.72. Ultimately, zafirlukast was determined as the most promising drug for repurposing [101].

Exploring drug–target interactions (DTIs) is a critical step in detecting drug actions and conducting drug repurposing. El-Behery et al. proposed a DTI prediction model that specifically integrates protein sequence and structured data [103]. The model uses the physical and chemical properties of protein amino acid sequences to obtain features and adopts encoding techniques to extract features from drug SMILES (Simplified Molecular Input Line Entry System) strings. Various machine learning techniques, deep learning techniques, and ensemble learning techniques are then applied to predict the interactions between drugs and target proteins in human cells. By using proteins affected by COVID-19 infection in human cells, they discovered potential drugs that are suitable for repurposing. For example, they predicted a 100% probability of interaction between ACE2 protein and DB00691 and DB05203 [103].

The primary challenge in drug repurposing lies in diagnosing and identifying unique drug–disease relationships [104,105]. Multiple AI algorithms can greatly facilitate drug repurposing and utilization during the COVID-19 era. Mohanty et al. used the Repurpose Drug Database and Open Chemical/Drug Database as inputs for their model, and then used machine learning, deep learning, RNNs, CNNs, and deep belief network algorithms to rapidly and accurately screen and output the desired drugs [106]. This technology enables drug repurposing without the need for initial and toxicity testing, allowing modified drugs to be used directly in late-stage treatment.

Phenotype-based compound screening, which uses gene expression profiles, offers advantages over target-based drug discovery and plays a crucial role in COVID-19 drug development [102,107]. Researchers have followed a mechanism-driven neural network approach called DeepCE, which incorporates graph neural networks and multi-head attention mechanisms [102]. This approach models the relationships between chemical substructures and genes, as well as gene–gene interactions, to predict differential gene expression profiles influenced by novel chemical entities (Fig. 5B). Additionally, the researchers extracted valuable information from the L1000 dataset to enhance the data. This method was applied to repurpose drugs for COVID-19. The team successfully identified 10 novel lead compounds that align with the clinical evidence, including chloramphenicol and cyclosporine [102].

### 6.2 Drug screening

In the search for potential small-molecule inhibitors against SARS-CoV-2, researchers have used neural networks to expedite virtual screening processes [108,109]. One noteworthy approach is the development of ChemAI, a deep neural network known as SmilesLSTM-ChemAI, created by Hofmarcher et al. [110]. This innovative model was trained using comprehensive drug databases such as ChEMBL, ZINC, and PubChem. By predicting biological outcomes such as target binding, inhibition, and toxicity, ChemAI ranks compounds based on their potential as inhibitors of SARS-CoV-2. Remarkably, this method facilitated the prediction of approximately one billion molecules. Subsequently, a library of potential SARS-CoV-2 inhibitors was generated, with compounds prioritized according to their inhibitory effects on SARS-CoV-2 proteases, potential toxicity, and proximity to known active compounds. Finally, this list was narrowed down to 30,000 compounds for further biological assay [110].

Ge et al. developed a framework for predicting effective candidate drugs against coronaviruses [111]. They applied machine learning and statistical analysis methods to create a data-driven drug repurposing framework that systematically integrated and mined extensive knowledge graphs, literature, and transcriptomic data to discover potential anti-SARS-CoV-2 candidate drugs. Through this framework, they identified poly (ADP-ribose) polymerase 1 inhibitor cvl218 as a potential therapeutic agent. In vitro experiments demonstrated the effective inhibitory activity of CVL1218 against SARS-CoV-2 replication, thereby validating the effectiveness of the framework in COVID-19 drug discovery [111].

Deep learning-based phenotypic omics profiling can unveil potential COVID-19 drugs [112]. Michael et al. used deep learning-based cellular morphological analysis to develop a "phenotypic omics" platform capable of identifying immunomodulators, toxins, pathogens, and genetic perturbations, as well as dose-dependent and high-dimensional relationships of small and large molecules [113]. This platform revealed a phenotypic model of active SARS-CoV-2 infection and COVID-19-associated cytokine storms. The authors discovered that Janus kinase inhibitors can effectively mitigate severe cytokine storm phenotypes, demonstrating the great potential of this mechanism within a complex immune cascade background [113].

## 7 Discussion and perspective

In this review, we summarized the innovative applications of AI methodologies in combating the COVID-19 pandemic. Advanced machine learning and deep learning models have shown tremendous potential in tackling critical challenges posed by this public health crisis. Specifically, AI-based predictive analytics enable accurate forecasting of disease spread trajectories and patient outcomes using clinical, epidemiological, and omics data. Deep neural networks facilitate rapid diagnosis based on medical images [114]. Intelligent systems incorporating risk assessment, decision support, and social sensing aid in pandemic control and shaping public health policies. Additionally, virtual screening powered by AI can augment therapeutic drug discovery and repurposing opportunities [115]. As highlighted in this review, the use of AI to fight COVID-19 is still in its early stages. Considerable progress has been made in the areas of prediction, detection, and drug discovery. However, predictive systems warrant further validation with real-world evidence. Diagnostic algorithms should evolve from binary classification to quantification of infection severity [116,117]. Although structural biology and bioinformatics models have identified various drug candidates, extensive clinical trials are needed to evaluate their safety and efficacy [118].

The reviewed studies demonstrate the great potential of AI in tackling the enormous challenges that have arisen as a result of the COVID-19 pandemic. Advanced machine learning and deep learning algorithms have enabled accurate prediction of disease spread, rapid diagnosis using medical images, data-driven policy making, and accelerated drug discovery. Multiple research teams have reported improved performance of AI models compared with traditional statistical methods in areas such as forecasting, detection, risk assessment, and drug repurposing.

Although AI has shown tremendous promise during the COVID-19 pandemic, there remain substantial challenges in translating proofs of concept into real-world impacts. A major limitation is a lack of the large, high-quality, standardized datasets required for developing robust AI models, especially early during the pandemic when reliable testing was absent [119], [120], [121]. Variations in demographics, protocols, and data formats can also constrain model generalization across different populations and settings [122]. Additionally, integration with existing clinical workflows and legacy information technology systems poses barriers to adoption, compounded by a lack of expertise in AI among frontline health care workers [123,124]. There are outstanding concerns regarding scalability, cost-effectiveness, and unfair biases when AI is deployed at scale. The “black box” nature of deep learning models hampers interpretability and accountability regarding AI-based decisions [125,126]. All these challenges are magnified by the urgency and unpredictability of the pandemic response, where reliance on inaccurate predictions or recommendations from immature AI could endanger human lives and undermine public trust. Thoughtful solutions embracing not just technical but also clinical, ethical, and social perspectives will be key to maximizing the benefits of AI for COVID-19 management.

When applying AI to address challenges in health care and biomedicine, it is crucial to assess various modeling approaches to determine the most effective methods [127], [128], [129]. For instance, in the context of AI-driven drug discovery and repurposing, researchers have conducted comparative evaluations of different machine learning and deep learning techniques. In one study, multiple algorithms were benchmarked to predict drug–target interactions using a dataset comprising over 460,000 compound-protein pairs [130]. These algorithms encompassed similarity-based methods, matrix factorization, graph convolutional networks (GCNs), and gradient boosting DTs. The GCN model had the highest performance, achieving an AUC of 0.965. It outperformed similarity-based methods by 3%–13% and other methods by 1%–7%. The effectiveness of the GCN model was attributed to its capacity to incorporate topological information from graphs and learn intricate connectivity patterns. When it comes to virtual screening for identifying bioactive compounds, deep learning generally surpasses other machine learning approaches. A comprehensive evaluation of 15 datasets revealed that deep neural networks achieved an average AUC 13% higher than those of random forests [131]. The advantages of deep neural networks lie in their ability to recognize complex feature representations and model interdependencies between input molecules and targets. However, interpretability remains a challenge [120]. In summary, comparative assessments offer practical insights into the tradeoffs and benefits of different AI techniques in health care applications. Further evaluation is necessary as innovative methods continue to emerge. Nevertheless, establishing head-to-head benchmarks using standard biomedical datasets can guide appropriate algorithm selection and optimize performance.

Finally, ethical risks surrounding privacy, fairness, and accountability must be addressed, especially when AI is used to guide high-stakes decisions like hospitalization triage [132,133]. Transparency regarding data provenance and model features is needed to build public and provider trust. Rigorous testing for biases and continuous monitoring of outcomes are imperative to avoid disproportionate impacts on marginalized communities [134,135]. Ultimately, AI should augment but not replace human expertise and experience during pandemic response.

Moving forward, multidisciplinary collaborations between computer scientists, biomedical researchers, and public health experts are crucial in developing robust, trustworthy, and socially responsible AI solutions with respect to COVID-19 as well as future pandemic threats. Advances in computational methods should be paired with a deep understanding of epidemiology, virology, health systems, and ethical implications. Beyond the sandbox of scientific publications, participatory design processes engaging diverse stakeholders will be instrumental in creating human-centered AI systems that solve real-world problems and improve population outcomes. Additionally, hybrid AI approaches combining the strengths of different techniques could enhance predictive power and applicability. Democratizing access to high-quality biomedical data and models would spur further innovations [136]. Detailed protocols and benchmarks are needed to fairly compare emerging methods. More importantly, centering human needs and values is vital as AI becomes increasingly involved in health care decisions.

In summary, translating promising AI applications into real-world impacts necessitates robust advances across technological, clinical, ethical, and implementation domains. Beyond innovations in algorithms and data, cultivating partnerships, infrastructure, evidence, and trust are all critical enablers of unlocking AI's potential to guide the global community through the COVID-19 pandemic and beyond.

## Funding

This research did not receive any specific grant from funding agencies in the public, commercial, or not-for-profit sectors.

## Author contributions

C.R.L.: Conceptualization, Methodology, Writing – original draft, Writing – review & editing. W.Q.G.: Conceptualization, Methodology, Writing – original draft, Writing – review & editing. X.Y.Y.: Conceptualization, Methodology, Writing – original draft, Writing – review & editing. L.L.: Writing – review & editing. X.L.H.: Writing – review & editing. S.M.L.: Writing – review & editing. L.Z.: Supervision, Funding acquisition.

## Acknowledgments

We would like to thank all those who have advised and helped us.

## Declaration of competing interest

The authors declare that they have no known competing financial interests or personal relationships that could have appeared to influence the work reported in this paper.
